# Poster Session I - A117 IMPACT OF PATIENT AND PHYSICIAN GENDER ON PHYSICIAN ASSESSMENT OF IBD REFERRALS

**DOI:** 10.1093/jcag/gwaf042.117

**Published:** 2026-02-13

**Authors:** J H Chow, G Wang, L Targownik

**Affiliations:** University of Ottawa, Ottawa, ON, Canada; Gastroenterology, University of Toronto, Toronto, ON, Canada; Gastroenterology, University of Toronto, Toronto, ON, Canada

## Abstract

**Background:**

Physicans’ implicit gender biases impact clinical practice and may be amplified if patients have co-existent mental health conditions. Gender biases in gastroenterology referrals have not been characterized, which could lead to disparities in triage and endoscopy. Patients with inflammatory bowel disease (IBD) often experience diagnostic delays, likely due to abdominal symptoms that may initially appear indistinguishable from irritable bowel syndrome. Therefore, we sought to assess how patient and physician gender, as well as mental health history, influence how physicians rate the likelihood of IBD in a patient referred with ambiguous gastrointestinal symptoms.

**Aims:**

To evaluate whether IBD likelihood scores differ between patient gender, mental health history, and physician gender.

**Methods:**

We conducted a between subjects randomized survey of North American gastroenterology physicians and fellows to review a hypothetical patient with features equivocal for Crohn’s Disease. Each participant was randomized to review male or female case vignettes which were otherwise identical, with or without a history of mental health comorbidities. Participants were located through gastroenterology organizations and snowball recruitment. Demographics and perceived likelihood of IBD (continuous scale 1-100) were collected. Descriptive statistics were used for demographics, and continuous variables used Welch’s t-test and two-way ANOVA for interaction effects, with *p*<0.05 considered as statistically significant.

**Results:**

Ninety-five physicians completed the survey (56% practicing, 44% in-training; 55% male, 44% female, 1% non-conforming). Male vignettes were perceived to have a significantly higher likelihood of IBD than females (mean difference= -7.8, *p*= 0.047). No differences were observed with mental health comorbidity (mean difference = +4.2, *p*= 0.29). Two-way ANOVA confirmed a main effect of patient sex (*F*(1,91)=4.11, *p*= 0.046), but not for mental health (*p*= 0.35) nor an interaction between these factors (*p*= 0.34). Incorporating physician gender in a secondary ANOVA, there were no significant effects (Table 1).

**Conclusions:**

This is the first study to directly survey gastroenterologists to explore whether patient or physician gender impacts clinician triage and perception of IBD likelihood, with significantly higher ratings in male than equivocal female vignettes. Future work should examine contributing factors to gender-related diagnostic bias and decision-making in clinical practice.

**Funding Agencies:**

Mount Sinai Hospital Resident Research Grant

**A117 Table 1:** Likelihood of IBD

Comparisons	Mean (SD)	Effect (95% CI or *F statistic*)	p-value
**Male vs female patients**	27.8 (22.2) vs 20.0 (14.8)	-7.8 (-15.6, -0.1)	0.047^*^
**Mental health vs none**	21.6 (18.5) vs 25.8 (20.1)	+4.2 (-3.6, 11.9)	0.29
**Gender x mental health**		F(1,91)=4.11 (gender); F = 0.87 (mental health); F = 0.91 (interaction)	0.046^*^ (gender); 0.35 (mental health); 0.34 (interaction)
**Physician x patient gender**		F(1,90)= 0.71 (physician gender); F = 3.00 (patient gender); F = 0.03 (interaction)	0.40 (physician gender); 0.087 (patient gender); 0.87 (interaction)

* significant values p < 0.05

